# Encrypted Three-dimensional Dynamic Imaging using Snapshot Time-of-flight Compressed Ultrafast Photography

**DOI:** 10.1038/srep15504

**Published:** 2015-10-27

**Authors:** Jinyang Liang, Liang Gao, Pengfei Hai, Chiye Li, Lihong V. Wang

**Affiliations:** 1 Optical Imaging Laboratory, Department of Biomedical Engineering, Washington University in St. Louis, Campus Box 1097, One Brookings Drive, St. Louis, Missouri 63130, USA; 2 Department of Electrical and Computer Engineering, University of Illinois at Urbana-Champaign, 405 North Mathews Avenue, Urbana, Illinois 61801, USA

## Abstract

Compressed ultrafast photography (CUP), a computational imaging technique, is synchronized with short-pulsed laser illumination to enable dynamic three-dimensional (3D) imaging. By leveraging the time-of-flight (ToF) information of pulsed light backscattered by the object, ToF-CUP can reconstruct a volumetric image from a single camera snapshot. In addition, the approach unites the encryption of depth data with the compressed acquisition of 3D data in a single snapshot measurement, thereby allowing efficient and secure data storage and transmission. We demonstrated high-speed 3D videography of moving objects at up to 75 volumes per second. The ToF-CUP camera was applied to track the 3D position of a live comet goldfish. We have also imaged a moving object obscured by a scattering medium.

Three-dimensional (3D) imaging technologies have been extensively studied for many years, going back to the “mirror stereoscope” invented by Sir Charles Wheatstone in 1838^1^. Since then, 3D imaging techniques have been used in many applications, including remote sensing, biology, and entertainment^2^,^3^,^4^. In the past decade, they have also been widely employed in the safety and national security communities^5^, in applications such as biometrics^6^,^7^, under-vehicle inspection^8^,^9^, and battlefield evaluation^10^,^11^. These applications impose demanding requirements—the 3D images must be captured and transmitted to users in a secure and fast manner.

The scattered photons from the object carry a variety of tags, such as emittance angle and time-of-flight (ToF), which convey the 3D surface information. Using these photon tags, numerous 3D imaging techniques have been developed, including structured-illumination^12^,^13^, holography^14^, streak imaging^15^,^16^, integral imaging^17^, multiple camera or multiple single-pixel detector photogrammetry^18^,^19^, and ToF detection^20^. Among these techniques, holography has demonstrated secure 3D surface imaging. Holography-based encryption is optically performed by using the diffraction pattern of a pseudo-random phase or amplitude mask^14^. This mask acts as the decryption key in reconstructing images of the 3D object from different viewing angles. However, as an interference-based approach, holographic imaging system is sensitive to motion during the relatively long exposure, which can severely degrade image quality.

ToF detection is another commonly used approach for 3D imaging. By measuring the ToF of a light signal between the object and the detector, the depth of the 3D object can be quantified. Among developed ToF detectors are the Microsoft Kinect^TM^ sensor^21^ for Xbox One and the single photon avalanche diode detector^22^. Although having largely improved the detection sensitivity, these ToF detectors acquire 3D images through multiple measurements, which limits applications for imaging fast-moving 3D objects.

To mitigate motion distortion in 3D images, single-shot ToF detection is an attractive approach. Among developed techniques^21^,^22^,^23^,^24^,^25^,^26^,^27^, the 3D Flash LIDAR camera^26^ has gained the most attention. This device can collect the entire spatiotemporal datacube within a single camera exposure, with an imaging speed up to 30 Hz^26^. However, the best achievable depth resolution is limited to ~10 cm^27^. In addition, the 3D images are first recorded on the sensor, followed by data compression and encryption for transmission^28^. Therefore, the sensor itself suffers from a heavy data load. In addition, the separate recording, compressing, and encrypting processes create a security loophole in the system.

To overcome these limitations, we present single-shot encrypted 3D imaging using compressed ultrafast photography (CUP). As a novel ultrafast imaging technique, CUP captures transient events with picosecond temporal resolution in a single snapshot^29^. Herein, we integrated CUP with active illumination and constructed a new ToF-CUP camera for snapshot volumetric imaging. Compared with previous techniques^30^,^31^,^32^,^33^, our system has three major advantages. First, the united process incorporating the encryption of depth data with the compressed acquisition of 3D data protects the security of private content. Second, each volumetric image is stored as a 2D snapshot in the measurement, which significantly reduces the data acquisition load on the sensor and therefore allows faster data communication to users. Third, the ToF-CUP camera can capture 3D images with a rate up to 75 volumes per second with a 1-cm depth resolution, outperforming the state-of-the-art single-shot 3D cameras. In the following, we will describe the principle of our approach, present the system prototype, and demonstrate encrypted 3D videography by imaging various moving 3D objects.

## Operation Principle

The principle of CUP has been detailed in our recent publication^29^. Here, we provide CUP with active illumination to detect photons backscattered from a 3D object. For collocated illumination and detection, the round-trip ToF signal carries information about the depth, *z*, relative to the point of light incidence on the object’s surface, which can be recovered by


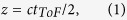


where 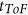 is the ToF of received photons, and *c* is the speed of light. The factor of two in the denominator on the right side of Eq. 1 accounts for the round-trip flight of photons.

A collimated laser beam illuminates the 3D object having intensity reflectivity *R*(*x*, *y*, *z*). The backscattered light signal from this 3D object, 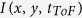, enters the ToF-CUP system. The depth information of the 3D object is conveyed as the ToF of the backscattered light signal. Mathematically, this process can be described by


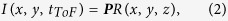


where ***P*** is a linear operator for light illumination and backscattering. Considering that the scattering is a linear process, 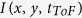 is linearly proportional to *R*(*x*, *y*, *z*). Our system then images this 3D object in three steps. First, the collected photons are spatially encrypted with a pseudo-random binary pattern, in which each pixel is set to either on or off. This pattern also acts as the decryption key to unlock and retrieve the image of the 3D object. Second, a streak camera temporally shears the ToF signal along the vertical direction. Third, the encrypted and sheared image is recorded on a CCD sensor in the streak camera via pixel-wise spatiotemporal integration. The optical energy measured at pixel 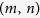 on the CCD, 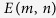, is related to the original 3D light intensity reflectivity, *R*(*x*, *y*, *z*), by


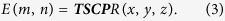


Here, ***T***, ***S***, and ***C*** are linear operators that represent spatiotemporal integration, temporal shearing, and spatial encryption, respectively. Equation 3 shows that the encryption process is inherently embedded in ToF-CUP.

Image decryption can be computationally performed by users who are granted the decryption key. If the 3D object is spatiotemporally sparse, 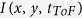 can be reasonably estimated by solving the inverse problem of Eq. 3 using compressed-sensing algorithms^34^,^35^,^36^,^37^,^38^,^39^. In our study, we choose the two-step iterative shrinkage/thresholding (TwIST) algorithm^34^, which minimizes a convex objective function given by


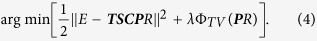


Here, 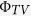 denotes the total-variation (TV) regularizer that encourages sparsity in the gradient domain during reconstruction^40^. The TwIST algorithm is initialized with a pseudo-random matrix of the discretized form of ***P**R* and then converged to a solution by minimizing the objective function in Eq. 4. The regularization parameter λ, which controls the weight of the TV regularizer, is adjusted empirically to provide the best for a given physical reality. Finally, 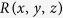 can be recovered given the linear relation between the backscattered light signal and the intensity reflectivity of the object. Further, in continuous shooting mode, the evolution of the 3D images over the “slow time”, 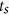, 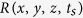, can be recovered by decrypting sequential snapshots. Here, the “slow time”, *t*_*s*_, relative to 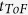, is defined as the time of capture of the imaged volume.

## System Configuration

**Figure 1:**
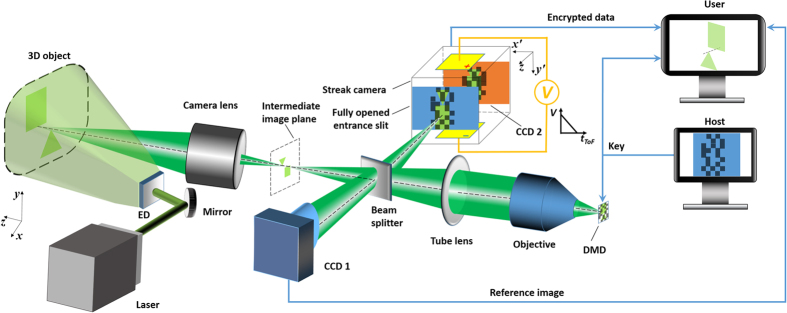
ToF-CUP system configuration. CCD, charge-coupled device; DMD, digital micromirror device; ED, engineered diffuser; *V*, sweeping voltage; 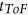, time-of-flight. Equipment details: camera lens, Nikon, *f* = 18–55 mm; CCD 1, Point Grey, FMVU-03MTM-CS; CCD 2, Hamamatsu, ORCA-R2; DMD, Texas Instruments, DLP LightCrafter 3000; engineered diffuser, Thorlabs ED1-S20-MD; laser, Attodyne, APL-4000; microscope objective, Olympus UPLSAPO 4×; streak camera, Hamamatsu C7700; tube lens, Thorlabs AC254-150-A.

The system schematic is shown in Fig. 1. A solid-state pulsed laser (532 nm wavelength, 7 ps pulse duration) is the light source. The laser beam passes through an engineered diffuser (ED) and illuminates a 3D object. The object is first imaged by a camera zoom lens (focal length 18–55 mm). Following the intermediate image plane, a beam splitter reflects half of the light to an external CCD camera (CCD 1 in Fig. 1), hereinafter called the reference camera, which records a reference 2D image of the 3D object. The other half of the light is transmitted through the beam splitter and passed to a digital micromirror device (DMD) by a 4-*f* imaging system consisting of a tube lens and a microscope objective (focal length 45 mm, numerical aperture 0.16). The total demagnification of the imaging system from the object to the DMD is ~46 ×.

To encrypt the input image, a pseudo-random binary pattern is generated by the host as the key and displayed on the DMD. Each encoded pixel in the binary pattern contains 3 × 3 DMD pixels (21.6 μm × 21.6 μm). The encrypted image is retro-reflected through the same 4-*f* system, reflected by the beam splitter, and imaged onto the fully opened entrance slit (~5 mm wide) of a streak camera. Deflected by a time-varying sweeping voltage, 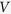, the light signal lands at various spatial locations on the *y’* axis according to its ToF. This temporally sheared image is recorded by an internal CCD sensor (CCD 2 in Fig. 1) in a single snapshot. This CCD sensor has 672 × 512 binned pixels (2 × 2 binning), and each encoded pixel is imaged by 3 × 3 binned CCD pixels. Finally, the encrypted data is transmitted to the user who decrypts the image with the key provided by the host.

The external CCD camera (CCD 1 in Fig. 1) is synchronized with the streak camera for each snapshot. An USAF resolution target is used to co-register images acquired by these two devices. Used as an intensity mask, the reference image is overlaid with the reconstructed 3D image to enhance the image quality. For each snapshot, the reconstructed 3D datacube contains *N*_*x*_ × *N*_*y*_ ×*N*_*z*_ = 150 × 150 × 350 voxels along the *x, y,* and *z* axes, respectively. In the *x-y* plane, this size gives a maximum imaging field-of-view (FOV) of 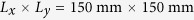. Given the collocated illumination and detection, the depth, 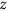, can be calculated by


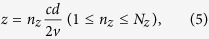


where *n*_*z*_ is the pixel index along the *z* axis, *d* is the CCD’s binned pixel size along the *y’* axis, and *v* is the shearing velocity of the streak camera. In our experiments, 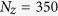, 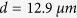, and *v* is set to 0.66 mm/ns. Therefore, the maximum depth range is 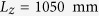.

## Results

**Figure 2:**
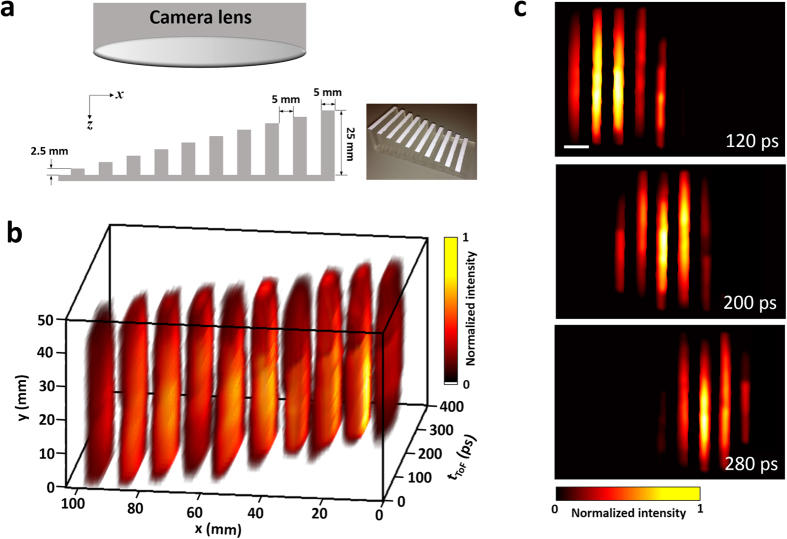
Quantification of ToF-CUP’s depth resolution. (**a**) Side view and photograph of the height-varying fin-pattern target used in the experiment. The ToF-CUP system was placed perpendicularly to the target base and collected backscattered photons from the target surface. (**b**) Reconstructed *x*, *y*, 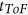 datacube representing the backscattered laser pulse intensity from the fins with different depths. (**c**) Representative *x-y* frames at 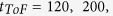 and 280 ps. Supplementary Video 1 shows the ToF snapshots of the *x-y* light distributions. Scale bar: 10 mm.

To quantify the system’s depth resolution, we imaged a 3D target with fins of varying heights (Fig. 2a). This target (100 mm × 50 mm along the *x* and *y* axes) was fabricated in-house using a 3D printer (Form 1+, Formlabs). Along the *x* axis, each fin has a width of 5 mm, and the height of the fins ascends from 2.5 mm to 25 mm, in steps of 2.5 mm. The imaging system was placed perpendicular to the target and collected the backscattered photons from the surface. Image reconstruction retrieved the ToF 2D images (Fig. 2b), and the corresponding movie of the ToF snapshots of the *x-y* light distributions is in Supplementary Video 1, which reveals the arrival sequence of photons backscattered by these fins. Three representative temporal frames at 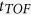 = 120, 200, and 280 ps are shown in Fig. 2c. In each frame, five fins are observed, indicating that the system’s depth resolution is approximately 10 mm.

**Figure 3:**
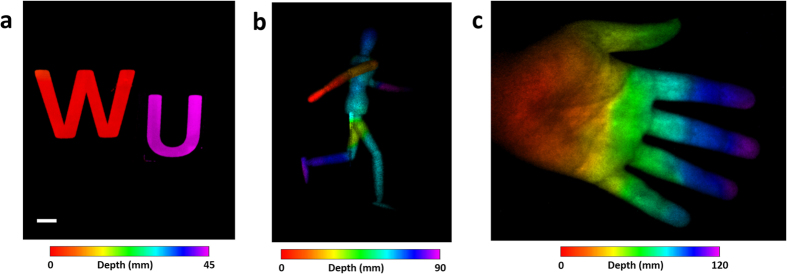
Depth-encoded ToF-CUP images of static objects. (**a**) letters “W” and “U” with a depth separation of 40 mm, (**b**) a wooden mannequin, and (**c**) a human hand. Scale bar: 10 mm.

To demonstrate ToF-CUP’s 3D imaging capability, we first imaged static objects (Fig. 3). Specifically, two letters, “W” and “U”, were placed with a depth separation of 40 mm (Supplementary Fig. 1a). The streak camera acquired a spatially-encrypted, temporally-sheared image of this 3D target in a single snapshot. The reference camera also directly imaged the same 3D target without temporal shearing to acquire a reference (Supplementary Fig. 1b). Using Eq. 5, the ToF signal was converted into depth information, and ToF-CUP reconstructed 3D *x, y, z* image of the target. For each pixel in the *x-y* plane, we found the maximum intensity in the *z* axis and recorded that coordinate to build a depth map. We color-encoded this depth map and overlaid it with the reference image to produce a depth-encoded image (Fig. 3a). For this object, the depth distance between the two letters was measured to be ~40 mm, which agreed with the true value. In addition, we imaged two additional static objects, a wooden mannequin and a human hand (Supplementary Fig. 1c,d). In both cases, we could successfully retrieve the depth information of the object using ToF-CUP (Fig. 3b,c). It is worth noting that the lateral resolution of the reconstructed datacube is ~0.1 line pairs per mm, and the reference images taken by the external CCD camera have a higher lateral resolution (~0.8 line pairs per mm). Because the depth-encoded image is produced by overlaying the depth map with the reference image, it has a lateral resolution limited by the reconstructed datacube.

**Figure 4:**
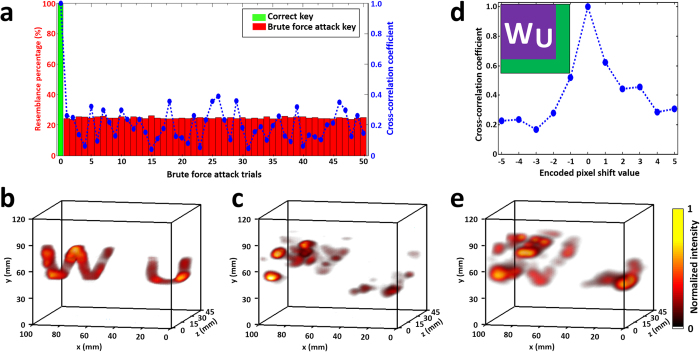
Security of the ToF-CUP camera against two types of decryption attacks. (**a**) Cross-correlation coefficients between the image decrypted using the correct decryption key and images using 50 brute force attacks with wrong pseudo-random binary masks. (**b**) 3D datacube of letters “W” and “U” decrypted using the correct decryption key. (**c**) As in (**b**), but using an invalid decryption key in the brute force attack (trial #26). (**d**) Cross-correlation coefficients between the reconstructed image decrypted using the correct decryption key and each image using a part of the correct decryption key with a different horizontal shift. Positive shift pixel numbers mean the key was shifted to the right of the acquired image, while negative numbers mean a shift to the left. The inset shows the relative positions of the letters “W” and “U” and the full decryption key (green). The part of the decryption key used in the shift security test is marked in purple. (**e**) As in (**b**) and (**c**), but using a part of the correct decryption key right shifted by one encoded pixel.

To verify the system’s encryption capability, we compared the image quality of the 3D datacubes reconstructed under two types of decryption attacks. The static 3D object “WU” was used in these tests. First, we imitated a brute force attack^28^, which attempted to guess the decryption key without any prior information. 50 pseudo-random binary masks were generated as invalid decryption keys. For each invalid key, we calculated its percentage of resemblance to the correct key. After the reconstruction, the cross correlations^41^ between the 3D datacubes based on these invalid keys and the one based on the correct key were calculated to quantify the reconstructed image quality (Fig. 4a). Without the valid decryption key, the reconstructed image quality is largely degraded, as reflected in the decreased correlation coefficients. For direct comparison, we show the reconstructed 3D datacube of the “WU” target produced by the valid and invalid keys (Fig. 4b,c). With the correct decryption key, the reconstructed image well resembles the object. The invalid decryption key, on the contrary, yields no useful information. In each attack, the reconstruction using the invalid key failed to retrieve the depth information, which demonstrated that our system is resistant to brute force attacks.

In addition, we tested the system’s performance when part of encryption key was known, but its position with respect to the encrypted image was unknown. To imitate this situation, a subarea (40 × 40 encoded pixels in the *x* and *y* axes) was selected from the full encryption key (50 × 50 encoded pixels in the *x* and *y* axes) as the decryption key (Fig. 4d inset). This decryption key was horizontally shifted by various numbers of the encoded pixels. For each shift, the reconstructed 3D datacube was compared with the correct reconstruction result to calculate the cross-correlation coefficient (Fig. 4d). The comparison shows that the reconstruction quality is sensitive to the relative position between the decryption key and the encrypted data (Fig. 4e), demonstrating that our system can protect the information in the 3D datacube even when part of the encryption key is leaked. We note that reconstructed datacubes from invalid decryption keys contain randomly distributed artifacts, some of which may have high intensity. These artifacts could affect the cross-correlation calculation. However, as shown in Fig. 4c,e, even with seemingly high cross-correlation coefficients, the reconstruction using invalid encryption keys does not resemble the original 3D object.

**Figure 5:**
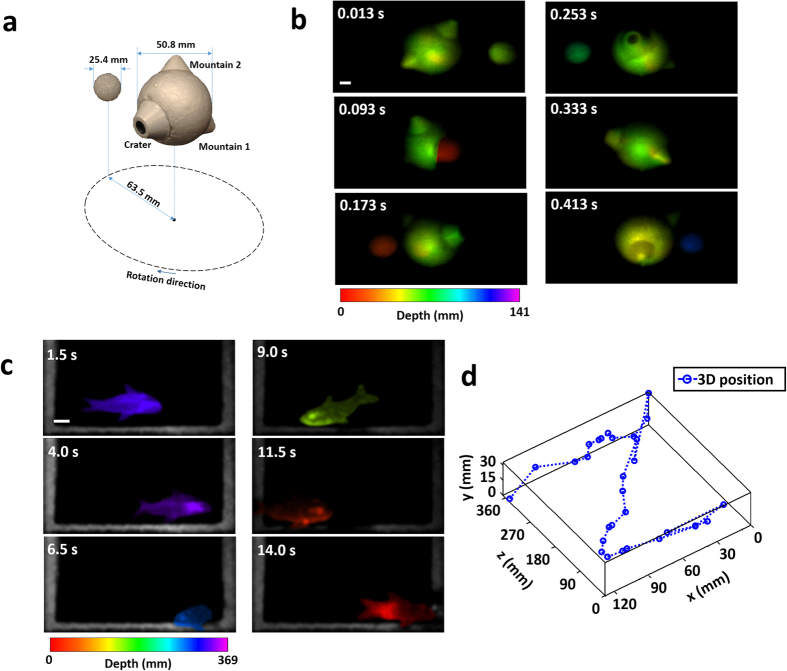
ToF-CUP of moving objects. (**a**) Experimental setup of two rotating balls. (**b**) Representative depth-encoded 3D images at six different slow-time points showing the relative depth positions of these two balls. The corresponding movie is in Supplementary Video 2. We also applied ToF-CUP imaging to a live comet goldfish. (**c**) Representative depth-encoded 3D images at six different slow-time points. (**d**) Trace of the 3D position of the fish. The corresponding movie is Supplementary Video 3. Scale bar: 10 mm.

To demonstrate ToF-CUP’s dynamic 3D imaging capability, we imaged a rotating object in real time (Fig. 5a). In the experiment, a foam ball with a diameter of 50.8 mm was rotated by a motorized stage at ~150 revolutions per minute. Two “mountains” and a “crater” were added as features on this object. Another foam ball, 25.4 mm in diameter, was placed 63.5 mm from the larger foam ball and rotated concentrically at the same angular speed. The ToF-CUP camera captured the rotation of this two-ball system by sequentially acquiring images at 75 volumes per second. Once each image was reconstructed to a 3D *x, y, z* datacube, these datacubes formed a time-lapse 4D *x, y, z, t*_*s*_ datacube. Figure 5b shows representative depth-encoded images at six different slow-time points, which reveals the relative depth positions of these two balls. The experimental setup of this moving 3D object and a reconstructed movie of its full-cycle rotation are shown in Supplementary Video 2.

To apply ToF-CUP’s dynamic 3D imaging capability to biological applications, we imaged a swimming comet goldfish (*Carassius auratus*). The ToF-CUP camera acquired 3D images at two volumes per second to capture the fish’s relatively slow movement over a sufficiently long time. Figure 5c shows six representative depth-encoded images of the fish. By tracing the centroid of each reconstructed datacube, we demonstrated 3D spatial position tracking of the fish (Fig. 5d). In this representative example, the ToF-CUP camera reveals that the fish first stayed at the rear lower left corner and then moved toward the right, after which it started to move toward the front wall of the fish tank. Supplementary Video 3 shows its full motion. In dynamic 3D imaging experiments, the external CCD camera was operated at a relatively long exposure time to tolerate relatively weak backscattered light. As a result, the movement of objects blurred the reference image. In contrast, because the exposure time of the streak camera is on the nanosecond level, the movement of the object did not noticeably affect the reconstructed datacube. Hence, the lateral and depth resolutions in the reconstructed images are not degraded.

**Figure 6:**
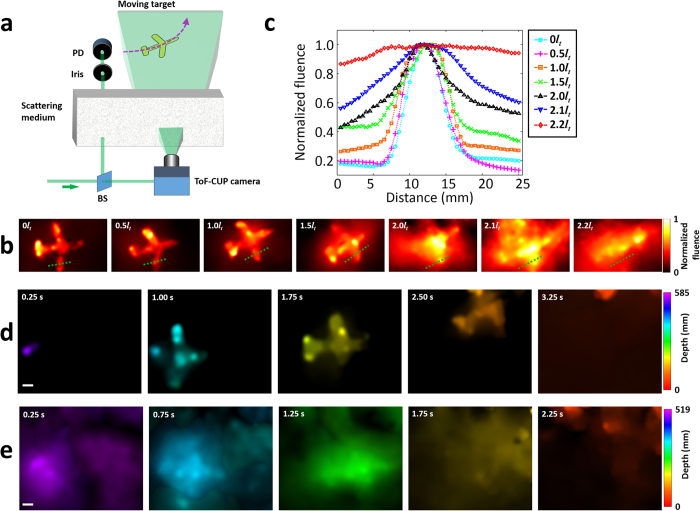
ToF-CUP imaging of an object moving behind a scattering medium. (**a**) Experimental setup. BS, beam sampler; PD, photodiode detector. The airplane moves from the lower left to the upper right, as well as toward the ToF-CUP camera in the depth direction. The trajectory of the airplane-model target is marked by the magenta dashed line with an arrow. (**b**) Projected images of the airplane-model target acquired at various scattering thicknesses of the scattering medium, where the projection was achieved by summing over the *x, y, z* datacube voxels along the *z* axis. (**c**) Comparison of the normalized fluence profiles of the airplane wing along the green dotted lines in (**b**). (**d**) Representative depth-encoded ToF-CUP images of an airplane target moving behind a scattering medium with an equivalent scattering thickness of 1.0*l*_*t*_. (**e**) As in (**d**), but with an equivalent scattering thickness of 2.1*l*_*t*_. The corresponding movie is Supplementary Video 4. Scale bar: 10 mm.

To explore ToF-CUP’s imaging capability in a real-world environment, we imaged an object moving behind a scattering medium that was composed by adding various concentrations of milk to water in a tank. The experimental setup is illustrated in Fig. 6a. Specifically, the incident laser beam was first de-expanded to ~2 mm in diameter. A beam sampler reflected a small fraction of the energy of the beam toward the tank. After propagating through the scattering medium, the transmitted beam passed through an iris (~2 mm in diameter). Then, the transmitted beam was measured by a photodiode detector to quantify the scattering level in the medium, which is presented as the equivalent scattering thickness in units of the mean free path (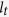). The rest of the incident laser beam was sent through the beam sampler and reflected by a mirror to an engineered diffuser (ED in Fig. 1), which generated wide-field illumination of a moving airplane-model target behind the scattering medium. This manually operated airplane-model target moved in a curved trajectory illustrated in Fig. 6a.

The ToF-CUP camera imaged this moving object through the scattering medium with various scattering thicknesses. To quantitatively compare the image quality, we selected a representative reconstructed 3D *x, y, z*, image at a single slow-time point for each scattering thickness, and summed over the 3D image voxels along the *z* axis. The resultant projected images are shown in Fig. 6b. In addition, the intensity profile of a cross section of the airplane wing is plotted under these conditions in Fig. 6c. The image contrast decreases with increased scattering in the medium and finally vanishes when the scattering thickness reaches 2.2*l*_*t*_. Figure 6d,e show representative images of this moving airplane target at five different slow-time points with two scattering thicknesses (1.0*l*_*t*_ in d and 2.1*l*_*t*_ in e), which record that the airplane-model target moved from the lower left to the upper right, as well as toward the ToF-CUP camera in the depth direction. Although scattering causes loss of contrast and features in the image, the depth can still be perceived. The corresponding movie is Supplementary Video 4. Due to the manual operation, the speed of the airplane-model target was slightly different in each experiment. As a result, the recorded movies with two scattering thicknesses (1.0*l*_*t*_ and 2.1*l*_*t*_) have different lengths, and so have the selected representative images in Fig. 6d,e.

## Discussion

Besides security, ToF-CUP offers the advantage of more efficient information storage and transmission because data is compressed during acquisition. ToF-CUP compresses a 3D datacube with 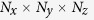 voxels to a 2D encrypted image with 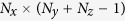 pixels. The data compression ratio can therefore be calculated as 
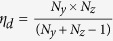
. With the current setup 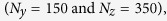 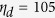. Therefore, ToF-CUP can potentially improve the data transmission rate by over two orders of magnitude. However, compared with optical bandwidth-limited images, the implementation of ToF-CUP degrades the spatial resolutions by factors of 1.8 and 2.2 along the *x* and *y* axes^29^. In addition, the depth resolution is degraded by 3.3 along the *z* axis, compared to the streak camera’s native resolution in resolving a ToF signal. Thus, regarding actual information content, we can estimate the data compression ratio by 
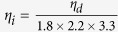
. For the current system, 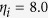.

Currently, the ToF-CUP camera’s performance is mainly restricted by the speed of the imaging devices and the illumination laser pulse energy. First, the streak camera’s sweeping repetition frequency and the CCD sensor’s readout speed limit the volume rate of the ToF-CUP camera. The current ORCA-R2 CCD^42^ in the streak camera has a full frame rate of 28 Hz, much lower than the streak camera’s sweeping repetition frequency of up to 1000 Hz^43^. Binning pixels or using a sub-array of the sensor could increase the readout rate at the expense of image resolution or FOV. Replacing the current CCD with an advanced high-speed CMOS sensor^44^ could largely increase the frame rate. In addition, ToF-CUP’s image reconstruction requires the DMD mask to be resolved in each encoded image at a given depth with a sufficient contrast-to-noise ratio (CNR). In our experiments, the minimal value of the required CNR was empirically determined to be 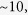 a value which yielded reliable reconstruction results. To provide enough illumination intensity, the maximal FOV at the object was limited to approximately 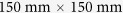 with the full laser energy (96.4 μJ per pulse). In addition, the maximum shearing velocity was held at 0.66 mm/ns in the streak camera, which corresponded to a spatial sampling interval of 3 mm along the *z* axis. With the current illumination intensity, a greater shearing velocity would reduce the number of collected photons per camera pixel and might result in an insufficient CNR.

The ToF-CUP camera can be potentially linked to a variety of future applications. For example, in terrestrial transportation, fog, haze, and dust pose severe potential threats to traffic safety. By integrating ToF-CUP with automobiles and aircraft, we could simultaneously track 3D positions of multiple objects in real time to avoid collisions caused by low visibility.

## Conclusions

In summary, we demonstrated encrypted 3D dynamic imaging using ToF-CUP. As a new type of ToF camera, ToF-CUP integrates the encryption of depth data with the compressed acquisition of 3D data in a single snapshot measurement, significantly enhancing information security and transmission efficiency. Our experiments demonstrated that the image of an original 3D object can be recovered only by users granted the decryption key. Moreover, from sequential image acquisition, we can track the 3D position of moving objects in real time. Finally, we demonstrated that ToF-CUP can image 3D moving objects obscured by a scattering medium. In the future, we plan to use a higher pulse-energy laser and a faster streak camera to further increase the imaging speed, FOV, and depth resolution.

## Additional Information

**How to cite this article**: Liang, J. *et al.* Encrypted Three-dimensional Dynamic Imaging using Snapshot Time-of-flight Compressed Ultrafast Photography. *Sci. Rep.* **5**, 15504; doi: 10.1038/srep15504 (2015).

## Supplementary Material

Supplementary Video 1

Supplementary Video 2

Supplementary Video 3

Supplementary Video 4

Supplementary Information
