# Supplementary material for: Encrypted Three-dimensional Dynamic Imaging using Snapshot Time-of-flight Compressed Ultrafast Photography
Source: Sci Rep. 2015 Oct 27;5:15504. doi: 10.1038/srep15504 (PMC4621413; doi:10.1038/srep15504)
Supplement: Supplementary Information [file srep15504-s5.pdf]

Encrypted Three-dimensional Dynamic Imaging using Snapshot Time-of-flight Compressed Ultrafast Photography

Jinyang Liang[†], Liang Gao[†], Pengfei Hai, Chiye Li, and Lihong V. Wang[*]

Optical Imaging Laboratory, Department of Biomedical Engineering, Washington University in St. Louis, Campus Box 1097, One Brookings Drive, St. Louis, Missouri 63130, USA

[*]Corresponding author: LHWANG@WUSTL.EDU

[†]These authors contributed equally to this work.

Supplementary Figure

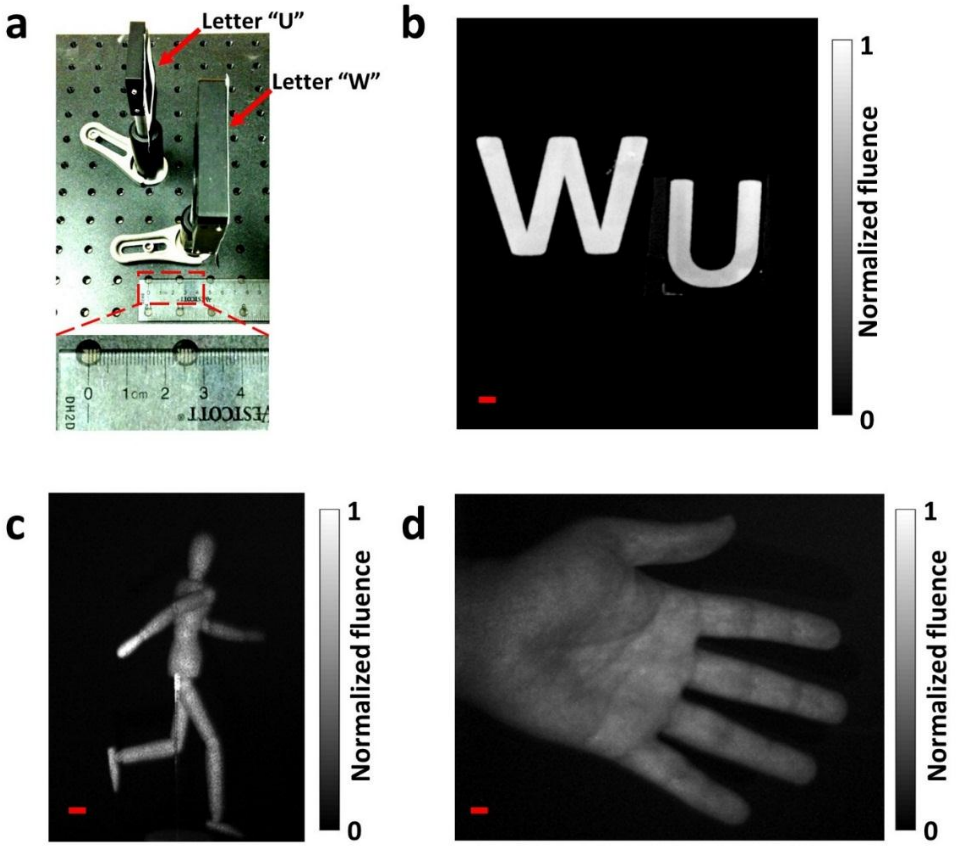

Supplementary Fig. 1. CCD images of static objects. (a) Side view of letters "W" and "U". Inset shows the depth separation is ~40 mm; Reference images of (b) letters "W" and "U", (c) a wooden mannequin, and (d) a human hand. Scale bar: 10 mm.

Supplementary videos

Video 1: Time-of-flight snapshots of the *x*-*y* light distributions from a fin pattern with varying heights.

Video 2: ToF-CUP imaging of a two-ball rotating target. Left: photograph of the experimental setup. Right: reconstructed movie.

Video 3: ToF-CUP imaging and tracking the 3D position of a live comet goldfish. Left: reconstructed 3D image of the fish versus time. Right: 3D position of the fish versus time.

Video 4: ToF-CUP imaging of an airplane target moving behind a scattering medium with equivalent scattering thicknesses of $1.0l_t$ (Left) and $2.1l_t$ (Right).
